# A New Era of Salvage-Line Treatment for Metastatic Colorectal Cancer: The Role and Clinical Significance of Circulating Tumor DNA

**DOI:** 10.3390/biom16040543

**Published:** 2026-04-07

**Authors:** Eiichiro Toyokawa, Akira Ooki, Eiji Shinozaki, Kaoru Yoshikawa, Keito Suzuki, Manabu Shiozawa, Shin Maeda, Kensei Yamaguchi, Hiroki Osumi

**Affiliations:** 1 Department of Gastroenterological Chemotherapy, Cancer Institute Hospital, Japanese Foundation for Cancer Research, Tokyo 135-8550, Japan; eiichirou.toyokawa@jfcr.or.jp (E.T.); akira.oki@jfcr.or.jp (A.O.); eiji.shinozaki@jfcr.or.jp (E.S.); kaoru.yoshikawa@jfcr.or.jp (K.Y.); keito.suzuki@jfcr.or.jp (K.S.); kensei.yamaguchi@jfcr.or.jp (K.Y.); 2 Department of Colorectal Surgery, Kanagawa Cancer Center, Yokohama 241-8515, Japan; shiozawa.0m702@kanagawa-pho.jp; 3 Department of Gastroenterology, Yokohama City University, Yokohama 236-0004, Japan; smaeda@yokohama-cu.ac.jp; 4 Department of Gastroenterology, Kanagawa Cancer Center, Yokohama 241-8515, Japan

**Keywords:** metastatic colorectal cancer, salvage line treatment, circulating tumor DNA, anti-epidermal growth factor receptor (EGFR) monoclonal antibody (mAb) rechallenge, FTD-TPI (trifluridine/tipiracil) ± BV (bevacizumab), multikinase inhibitors

## Abstract

The emergence of novel cytotoxic agents, multikinase inhibitors, and various antibody-based therapies has significantly expanded salvage therapy options for metastatic colorectal cancer (mCRC). Consequently, establishing the optimal treatment sequence for patients has become a formidable clinical challenge. Emerging evidence highlights the value of comprehensive biomarker assessment, particularly longitudinal monitoring of circulating tumor DNA (ctDNA), to capture dynamic molecular changes during treatment. Liquid biopsy-based technologies now enable real-time tracking of molecular alterations, supporting truly personalized therapeutic decision-making. Furthermore, prior treatment exposure and residual toxicities must be carefully considered to balance efficacy, safety, and quality of life. This review provides a comprehensive overview of the current salvage-line landscape for mCRC, discusses the clinical utility of ctDNA as a predictive and prognostic tool, and proposes integrated strategies to optimize therapeutic outcomes in the evolving era of precision medicine.

## 1. Introduction

Colorectal cancer (CRC) remains a major global health burden, characterized by high incidence and mortality rates. In 2022, an estimated 1,926,118 new cases and 903,859 deaths were reported, ranking CRC third in cancer incidence and second in cancer-related mortality globally [1]. Although the incidence rates of CRC among older adults have declined in high-income countries, its global burden continues to rise, particularly due to early-onset CRC in individuals younger than 50 years old and in developing countries [2].

Significant advances in early detection and surgical interventions have improved the outcomes of CRC. However, approximately 20–25% of patients present with metastatic disease at initial diagnosis [3,4], and an additional 20–50% develop metastases during the course of the disease [5]. Notably, the liver is the most common site of CRC metastasis, followed by the lungs, peritoneum, and distant lymph nodes [6]. Meanwhile, the treatment of metastatic colorectal cancer (mCRC) has advanced significantly in recent years, resulting in marked improvements in patient outcomes, with contemporary studies indicating a median overall survival (OS) of approximately 32–40 months [7]. These advances have been driven by three key factors: (i) increasingly refined biomarker-guided treatment selection, (ii) improved local therapies for liver and lung metastases, and (iii) introduction of novel systemic therapeutic agents [8,9]. For most patients, fluoropyrimidines, oxaliplatin, and irinotecan form the backbone of first- and second-line systemic therapy [10,11]. However, the management of patients who progress beyond these standard therapies remains a clinical challenge. In the salvage-line setting, defined as third-line or later-line treatment, regorafenib and trifluridine/tipiracil (FTD-TPI), were historically considered the principal options, albeit with modest survival benefits. More recently, the introduction of novel agents such as fruquintinib and a growing array of biomarker-driven treatments has expanded the therapeutic landscape for mCRC. Despite these advances, robust evidence defining the optimal treatment sequence for mCRC in salvage-line settings remains limited.

Circulating tumor DNA (ctDNA) has emerged as a minimally invasive diagnostic tool that provides real-time insight into the molecular profile of tumors [12,13,14]. ctDNA analysis enables dynamic monitoring of clonal evolution and the emergence—or potential regression—of resistance mutations, thereby offering a biological rationale for adaptive, individualized treatment strategies, including anti-epidermal growth factor receptor (EGFR) monoclonal antibodies (mAbs) rechallenge [15,16].

As the therapeutic options for mCRC continue to expand, establishing the optimal treatment sequence and identifying patients most likely to benefit from specific interventions have become increasingly important. However, the growing complexity and diversification of treatment strategies in the era of precision oncology underscore the need for an updated and integrated review to clarify optimal sequencing strategies and the role of biomarker-guided approaches. In this review, we summarize findings from pivotal clinical trials and the latest evidence regarding salvage-line treatments for mCRC. In addition, we discuss the utility of ctDNA-guided approaches for refined patient selection and therapeutic decision-making in the era of precision oncology, while providing perspectives on future treatment strategies in this rapidly evolving field.

## 2. Evolution of Salvage-Line Therapies

### 2.1. Anti-EGFR mAbs and the Significance of RAS Status

Anti-EGFR mAbs, such as cetuximab and panitumumab, were initially developed as salvage options for patients refractory or intolerant to standard cytotoxic chemotherapy. The phase III CO.17 trial, which included patients refractory or intolerant to fluoropyrimidines, irinotecan, and oxaliplatin, demonstrated that cetuximab significantly improved OS (median OS [mOS]: 6.1 vs. 4.6 months; hazard ratio [HR]: 0.77; *p* = 0.005) and progression-free survival (PFS) (median PFS: 1.9 vs. 1.8 months; HR: 0.68; *p* < 0.001) and while maintaining quality of life compared with best supportive care (BSC) [17]. Subsequent molecular analyses revealed that this efficacy was strictly limited to patients with *RAS* wild-type (WT) tumors [18]. The phase III study 20020408 showed that compared with BSC alone, panitumumab significantly improved PFS (median PFS [mPFS]: 8.0 vs. 7.3 weeks; HR: 0.54; *p* < 0.001) but not OS, likely due to crossover in 76% of patients who received BSC [19]. Molecular analyses showed that the PFS benefit was confined to patients with *KRAS* WT tumors, particularly those with *NRAS* and *BRAF* WT tumors [20]. Similarly, the phase III 20100007 trial confirmed that panitumumab provided a substantial OS benefit (mOS: 10.0 vs. 7.4 months; HR: 0.73; *p* = 0.0096), specifically in patients with *RAS* WT tumors [21]. These findings indicate anti-EGFR mAbs as the first agents to demonstrate a survival benefit over BSC in patients with *RAS* WT mCRC in the salvage-line setting, establishing *RAS* mutational status as an indispensable biomarker for treatment selection.

Combination therapies with irinotecan have been explored to further enhance treatment efficacy. The phase II BOND trial showed that cetuximab plus irinotecan yielded a superior objective response rate (ORR) (22.9% vs. 10.8%; *p* = 0.007) and prolonged PFS compared with cetuximab monotherapy (mPFS: 4.1 vs. 1.9 months; *p* < 0.001), suggesting that anti-EGFR mAbs could partially overcome irinotecan resistance [22]. Regarding the choice of anti-EGFR mAbs, the ASPECCT and WJOG 6510G trials demonstrated the non-inferiority of panitumumab to cetuximab in terms of PFS and OS; however, their safety profiles differed, with panitumumab associated with fewer infusion reactions but a higher incidence of hypomagnesemia [23,24].

Several studies have suggested that prior bevacizumab exposure may reduce the efficacy of cetuximab [25,26,27]. In the ASPECCT subgroup analyses, patients previously treated with bevacizumab appeared to benefit more from panitumumab. In a pooled analysis of the ASPECCT and WJOG 6510G trials, panitumumab significantly improved OS compared with cetuximab (mOS: 12.8 vs. 10.1 months; HR: 0.72; *p* = 0.0031) as well as PFS (mPFS: 4.7 vs. 4.1 months; HR: 0.79; *p* = 0.021) [28]. Therefore, efficacy, toxicity, and administration schedule should be considered in treatment selection, particularly for bevacizumab-pretreated patients.

Right-sided tumors are typically resistant to anti-EGFR mAb. However, in carefully selected patients requiring a high response, anti-EGFR mAb therapy may still be considered [5]. Exploratory analysis conducted in the phase III PARADIGM trial showed that in patients without resistance mutations (e.g., *RAS* or *BRAF* V600E), those treated with panitumumab plus mFOLFOX6 tended to exhibit longer OS than those treated with bevacizumab plus mFOLFOX6 (mOS: 38.9 vs. 30.9 months; HR: 0.82; *p* = 0.145) [29]. Similarly, a retrospective study identified *RAS*/*BRAF* V600E mutations—but not tumor sidedness—as independent adverse prognostic factors [30]. These findings suggest that anti-EGFR mAb therapy may still benefit selected patients with right-sided mCRC who are anti-EGFR–naïve and should be considered in the salvage-line setting.

In summary, the combination of anti-EGFR mAb with and without irinotecan is a salvage-line strategy especially for patients with *RAS* WT mCRC; however, its application remains strictly limited to molecularly selected populations.

### 2.2. Regorafenib

The limitation of anti-EGFR mAb therapies to *RAS* WT tumors highlighted a critical need for agents that demonstrate efficacy regardless of mutational status.

Regorafenib, an oral multikinase inhibitor targeting VEGFR1-3, TIE2, RAF1, BRAF, PDGFR, and FGFR, was developed to inhibit tumor angiogenesis and modulate the tumor microenvironment [31]. The phase III CORRECT trial, which included patients refractory or intolerant to standard therapies, demonstrated that regorafenib significantly improved OS compared with placebo (mOS: 6.4 vs. 5.0 months; HR: 0.77; *p* = 0.0052) [32]. The CONCUR trial corroborated these results in Asian populations (mOS: 8.8 vs. 6.3 months; HR: 0.55; *p* = 0.00016) [33]. Despite frequent reports of adverse events such as hand–foot skin reactions and fatigue, regorafenib has been established as a foundational salvage agent.

The ReDOS trial, a randomized phase II study of patients with mCRC, showed that weekly dose escalation of regorafenib (from 80 mg to 120 mg daily and then to 160 mg daily) improved treatment continuation, including a higher rate of starting cycle 3 (43% vs. 26%; *p* = 0.043), reduced discontinuations due to toxicity, and enhanced quality of life compared with the standard 160 mg starting dose [34].

These findings suggested that initiating treatment with dose reduction and proactively managing adverse events are crucial for maintaining treatment continuity.

### 2.3. Trifluridine/Tipiracil

Trifluridine/tipiracil (FTD-TPI) is an oral agent comprising trifluridine, a thymidine analog, and tipiracil, which inhibits its degradation [35]. In the global phase III RECOURSE trial, FTD-TPI demonstrated a significant survival advantage (mOS: 7.1 vs. 5.3 months; HR: 0.68; *p* < 0.001) and prolonged the time to performance status deterioration [36]. The TERRA trial confirm these findings in Asian patients. However, despite evidence indicating that FTD-TPI provides consistent disease stabilization and survival prolongation with a manageable safety profile, its limited capacity for tumor shrinkage prompted the development of more intensive combination regimens [37].

Analyses of the RECOURSE and J003 trials revealed that patients who developed chemotherapy-induced neutropenia (CIN) during the early phase of FTD-TPI treatment (cycles 1–2) exhibited significantly longer PFS and OS than those who did not or those in the placebo group [38]. CIN occurrence is correlated with FTD-TPI drug exposure, suggesting adequate systemic drug levels. These findings have been consistently reproduced in multiple real-world cohorts and retrospective analyses, in which grades 3–4 neutropenia emerged as an independent favorable prognostic factor [39,40]. Accordingly, neutropenia is recognized as an on-treatment biomarker of efficacy during FTD-TPI therapy.

In a subgroup analysis of the RECOURSE trial, patients harboring *KRAS* G12C mutations did not show any OS benefit compared with patients in the placebo group (HR: 0.97; *p* = 0.85) [41]. In addition, a meta-analysis of three randomized controlled trials—RECOURSE, TERRA, and J003—demonstrated that although patients with *KRAS* G12C mutations still showed prolonged OS, the magnitude of the benefit was attenuated [42]. Accordingly, *KRAS* G12C mutation is recognized as a biomarker associated with poor prognosis in patients undergoing FTD-TPI monotherapy.

Collectively, these findings suggest that FTD-TPI monotherapy can prolong OS, provided that appropriate dose modifications and supportive care are implemented.

### 2.4. Fruquintinib

Fruquintinib is a highly selective oral inhibitor of VEGFR-1, -2, and -3 designed to exert potent antitumor activity by inhibiting tumor-induced angiogenesis. Its distinct pharmacological profile, characterized by superior kinase selectivity compared with other multikinase inhibitors, minimizes off-target toxicities while maximizing VEGF pathway suppression [43,44].

The clinical efficacy of fruquintinib was first established in the Chinese phase III FRESCO trial, in which it demonstrated significantly improved OS (mOS: 9.3 vs. 6.6 months; HR: 0.65; *p* < 0.001) and substantially prolonged PFS (mPFS: 3.7 vs. 1.8 months; HR: 0.26; *p* < 0.001) compared with placebo [45]. These findings were subsequently validated on a global scale in the FRESCO-2 trial, which included heavily pretreated patients previously exposed to regorafenib or FTD-TPI. In this challenging context, fruquintinib maintained a consistent survival benefit (mOS: 7.4 vs. 4.8 months; HR: 0.66; *p* < 0.0001) with a manageable safety profile [46]. The global approval of fruquintinib has significantly expanded the therapeutic landscape of mCRC, providing a robust option for patients who have exhausted traditional lines of therapy. The Japanese subgroup analysis of the FRESCO-2 trial showed a higher incidence of grade ≥ 3 adverse events overall, as well as increased rates of hypertension, proteinuria, and hand–foot skin reactions of any grade, compared with Western populations [47]. Although these events were manageable, careful monitoring is warranted during therapy.

These findings establish fruquintinib as a valuable salvage-line option for mCRC, offering consistent survival benefits across diverse populations regardless of race, biomarker status, or prior treatment history, although careful toxicity monitoring remains essential.

## 3. FTD-TPI Plus Bevacizumab: An Emerging Standard of Care

As the synergy between cytotoxic agents and angiogenesis inhibition became increasingly evident, early-phase clinical studies explored the addition of bevacizumab to FTD-TPI to enhance therapeutic intensity. In the single-arm phase I/II C-TASK FORCE trial, FTD-TPI plus bevacizumab achieved a 16-week progression-free survival rate of 42.9% [48]. These findings led to the randomized phase II Danish trial, in which FTD-TPI plus bevacizumab significantly prolonged PFS compared with FTD-TPI monotherapy (mPFS: 4.6 vs. 2.6 months; HR: 0.45; *p* = 0.0015) [49]. Subsequently, the landmark phase III SUNLIGHT trial included patients regardless of mutational status and demonstrated that FTD-TPI plus bevacizumab significantly improved OS compared with FTD-TPI monotherapy (mOS: 10.8 vs. 7.5 months; HR: 0.61; *p* < 0.001) and PFS (5.6 vs. 2.4 months; HR: 0.44; *p* < 0.001), without a clinically meaningful increase in severe adverse events [50]. The efficacy of this regimen was consistent across various subgroups, including older patients and those with *RAS*-mutant tumors [51].

Notably, CIN has emerged as an on-treatment biomarker associated with the efficacy of FTD-TPI [38]. A single-center retrospective study indicated an association between severe neutropenia during early treatment cycles and prolonged survival in patients treated with FTD-TPI plus bevacizumab [52]. Furthermore, post hoc analyses of the SUNLIGHT trial demonstrated that neutropenia, including grade ≥ 3 neutrophil decrease, is associated with improved PFS and OS [53]. Alternative schedules designed to optimize tolerability and convenience, such as biweekly administration, have shown promising activity and safety in phase II studies (e.g., the BiTS and TAS-CC4 trials) [54,55]. A pragmatic, randomized phase III trial (the PRABITAS study) is currently underway to assess the non-inferiority of biweekly FTD-TPI plus bevacizumab [56].

FTD-TPI plus bevacizumab has been established as a preferred third-line strategy for a broad population of patients with mCRC. However, careful management of adverse events, such as neutropenia and proteinuria, is essential to optimized treatment continuity and outcomes.

A summary of the key clinical trials of conventional salvage-line therapies is presented in Table 1.

## 4. Molecularly Guided Therapies

In this context, molecularly guided therapies refer to treatment strategies tailored according to specific genomic alterations identified by tissue or ctDNA analysis, including *RAS* wild-type status, *ERBB2* amplification, *KRAS* G12C mutation, and *BRAF* V600E mutation.

### 4.1. Anti-EGFR mAb Rechallenge

The paradigm of precision oncology is predicated on accurate identification of molecular alterations that dictate sensitivity or resistance to targeted therapies. The molecular profile of mCRC is not static but evolves under the selective pressure of treatment [57,58,59]. Liquid biopsy is an indispensable, minimally invasive tool that can capture these dynamic genomic shifts in real time. Among various analytes, ctDNA is regarded as the most clinically informative component, allowing for longitudinal assessment of spatial and temporal tumor heterogeneity that tissue biopsies cannot easily replicate [60]. Through comprehensive molecular profiling, ctDNA has emerged as a central biomarker that supports clinical decision-making.

These applications span several clinical domains, with particular relevance in the early detection of disease recurrence and progression. ctDNA mutations have been shown to precede radiographic recurrence in both mCRC and postoperative CRC, highlighting its utility in the early identification of relapse and disease progression. Elevated baseline ctDNA levels are associated with poorer OS and PFS [61,62], supporting its role as a prognostic biomarker. Furthermore, multiple meta-analyses have demonstrated that ctDNA positivity after surgery or adjuvant therapy is an independent predictor of poor prognosis in CRC, significantly associated with increased recurrence risk and shorter relapse-free survival (RFS), underscoring its value in minimal residual disease (MRD) detection [63,64,65,66]. Cell-free DNA (cfDNA) has a very short half-life of approximately one hour, and ctDNA, as a tumor-derived fraction of cfDNA, dynamically reflects tumor burden and treatment response more effectively than conventional tumor markers or imaging modalities [67,68,69,70]. Increases in ctDNA levels during treatment are associated with poor prognosis, whereas early declines correlate with favorable outcomes in mCRC [71,72,73]. Moreover, ctDNA has been shown to outperform tissue biopsy in capturing tumor heterogeneity [74] and has robust evidence supporting its utility in identifying resistance mutations to anti-EGFR therapy [75,76]. Taken together, ctDNA serves as a clinically important biomarker in mCRC, encompassing early detection of recurrence and disease progression, prognostic stratification, treatment response monitoring, and identification of resistance mechanisms, thereby playing a central role in guiding clinical decision-making.

To support these clinical applications, various analytical platforms are currently employed for ctDNA detection, including polymerase chain reaction (PCR)-based assays and next-generation sequencing (NGS) approaches. Among PCR-based methods, droplet digital PCR (ddPCR) enables rapid and cost-effective quantification of known driver mutations and is well suited for longitudinal monitoring of specific alterations; however, its reliance on prior knowledge of target mutations limits its ability to assess tumor heterogeneity and detect novel resistance mechanisms [77,78]. In contrast, NGS-based approaches, including both targeted and non-targeted strategies, allow simultaneous evaluation of multiple genomic alterations and provide comprehensive molecular profiling from ctDNA [79,80].

The clinical utility of ctDNA is best exemplified by the strategy of anti-EGFR mAb rechallenge. Although most patients who initially respond to anti-EGFR therapy eventually develop acquired resistance [76]—primarily through the emergence of *RAS*, *BRAF*, or *EGFR* extracellular domain (EGFR-ECD) mutations [81,82,83]—these resistant clones often exhibit reduced fitness in the absence of the drug. Following a treatment-free interval, these mutant clones may decay exponentially, allowing *RAS* WT clones to re-emerge as the dominant population and potentially restoring sensitivity to anti-EGFR therapy [75,84,85,86].

Santini et al. conducted a prospective single-arm study of 39 patients with mCRC who had achieved clinical benefits from first-line cetuximab plus irinotecan therapy and later showed disease progression. After a median anti-EGFR mAb-free interval of 6 months, rechallenge with the same regimen yielded an ORR of 53.8% and mPFS of 6.6 months, providing proof-of-concept evidence of activity [87]. Similarly, the multicenter phase II CRICKET trial, which was conducted to evaluate third-line cetuximab rechallenge in *RAS*/*BRAF* WT mCRC, indicated an ORR of 21%. Treatment responses were observed only in patients without baseline ctDNA *RAS* mutations. In addition, ctDNA *RAS* WT status was associated with a longer PFS and a trend toward improved OS [88]. Similar findings were observed in the E-Rechallenge trial, post hoc analyses of the JACCRO CC-08 and CC-09 trials, and the phase II CAVE study, with ctDNA-defined *RAS*/*BRAF* WT status associated with prolonged survival [89,90,91]. Comparable results have also been reported in real-world retrospective cohorts [92]. Collectively, these data support the clinical utility of ctDNA-guided reassessment.

Findings from prospective ctDNA-guided trials further support this strategy. In the CHRONOS study, patients with tissue and ctDNA-confirmed *RAS*/*BRAF*/EGFR-ECD WT tumors achieved an ORR of 30% and a median PFS of 4.0 months, demonstrating the clinically meaningful activity of anti-EGFR mAb rechallenge in the third-line setting [93]. The randomized phase II CITRIC trial showed trends toward improved PFS and OS in patients treated with cetuximab plus irinotecan versus those treated with physician’s choice therapy. However, the primary endpoint of the study was not met; interpretation was influenced by inclusion of FTD-TPI plus bevacizumab in the control arm [94]. In contrast, the phase III FIRE-4 trial, in which ctDNA-guided selection was not mandatory, revealed no significant differences in survival between the rechallenge and control arms, likely reflecting both effective control-arm therapies and the absence of molecular selection [95].

**Figure 1:**
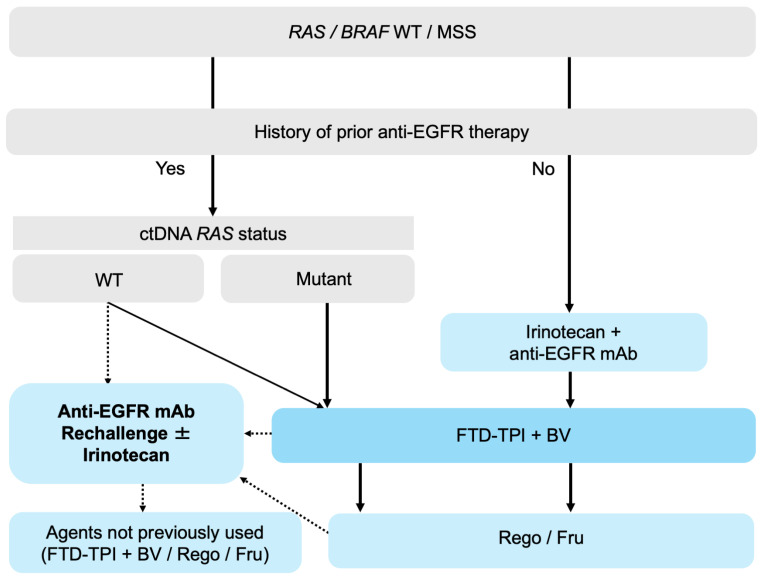
Treatment algorithm for anti-EGFR mAb rechallenge in *RAS*/*BRAF* WT mCRC. Abbreviations: mCRC, metastatic colorectal cancer; WT, wild-type; MSS, microsatellite stable; EGFR, epidermal growth factor receptor; mAb, monoclonal antibody; FTD-TPI, trifluridine/tipiracil; BV, bevacizumab; Rego, regorafenib; Fru, fruquintinib. Solid arrows indicate the preferred treatment pathway. Dashed arrows indicate alternative or context-dependent options.

Collectively, although the optimal positioning of anti-EGFR mAb rechallenge relative to FTD-TPI plus bevacizumab remains uncertain, it may be reasonably considered in carefully selected patients, particularly those with a tissue-confirmed *RAS* WT status, a prior favorable response to anti-EGFR therapy, ctDNA-confirmed *RAS* WT status immediately before rechallenge, an anti-EGFR–free interval of ≥4 months, a clinical need for tumor shrinkage, and limited suitability for angiogenesis inhibitors (Figure 1).

When performing ctDNA assessment, several important considerations warrant attention. First, given the short half-life of ctDNA, testing should ideally be performed immediately prior to rechallenge, as this timing allows for a more accurate reflection of the current tumor biology. Second, when *RAS* mutations are detected at a low VAF—particularly below 1%—false-positive results attributable to CHIP should be suspected, especially in elderly patients or those with prior chemotherapy exposure; in such cases, matched white blood cell sequencing alongside ctDNA testing is recommended to exclude CHIP-related mutations [96,97]. Third, no consensus definition currently exists for what constitutes a high ctDNA level, which complicates result interpretation [68]. Fourth, ctDNA shedding may be reduced in patients with low disease burden or specific metastatic sites—such as pulmonary, peritoneal, or brain metastases—potentially leading to false-negative results [98,99,100,101]. Finally, variability across assay platforms may affect the comparability and reproducibility of ctDNA measurements [102].

Accordingly, the key clinical trials of anti-EGFR mAb rechallenge are summarized in Table 2.

### 4.2. HER2-Positive mCRC: Emergence of Dual Blockade and Antibody–Drug Conjugates

In mCRC, human epidermal growth factor receptor 2 (HER2; *ERBB2*) gene amplification or overexpression is observed in approximately 2–3% of cases overall and in around 5% of *KRAS*/*NRAS*/*BRAF* WT tumors [103]. These tumors are often resistant to anti-EGFR therapy, establishing HER2 as a critical therapeutic target [104,105].

Several HER2-directred therapeutic strategies have been evaluated in mCRC, ranging from dual antibody blockade to include potent antibody–drug conjugates (ADCs). Regarding dual HER2 blockade, the HERACLES-A, MyPathway, and TRIUMPH trials demonstrated the efficacy of combining trastuzumab with lapatinib or pertuzumab, yielding ORRs ranging from approximately 30% to 32% [106,107,108]. Of note, the TRIUMPH trial validated the clinical utility of ctDNA for identifying HER2-positive candidates. Regarding ADCs, the DESTINY-CRC01 and CRC02 trials demonstrated that trastuzumab deruxtecan (T-DXd), a next-generation ADC, showed high activity in patients with pretreated HER2-positive mCRC [109,110]. The 5.4 mg/kg dose is now favored due to its balanced profile of encouraging efficacy (ORR 37.8%) and manageable safety, particularly in patients with interstitial lung disease. Furthermore, the MOUNTAINEER trial demonstrated that tucatinib plus trastuzumab, a dual HER2 blockade, achieved an ORR of 38.1% and a median OS of 24.1 months [111], leading to the evaluation of this combination as a first-line treatment in the ongoing MOUNTAINEER-03 trial [112].

**Figure 2:**
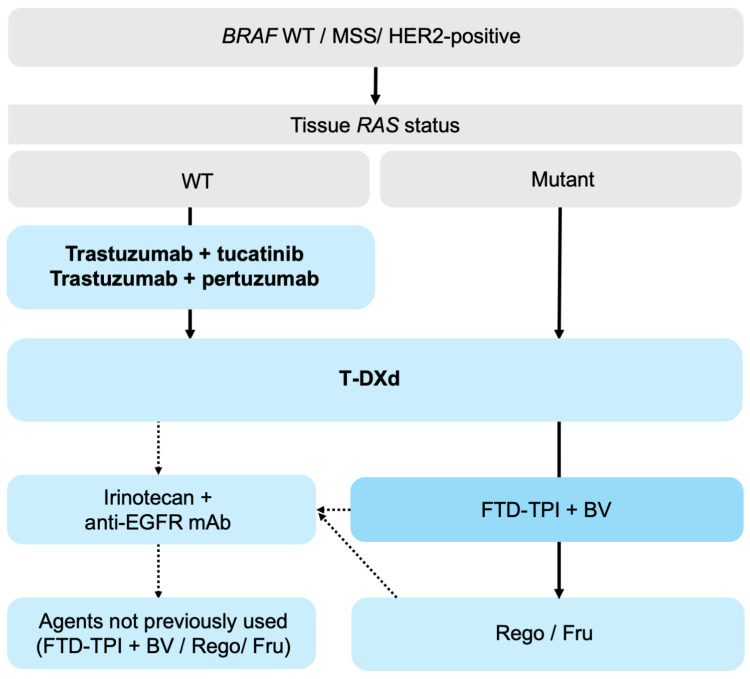
Treatment algorithm for HER2-positive mCRC. Abbreviations: mCRC, metastatic colorectal cancer; WT, wild-type; MSS, microsatellite stable; T-DXd, trastuzumab deruxtecan; FTD-TPI, trifluridine/tipiracil; BV, bevacizumab; Rego, regorafenib; Fru, fruquintinib. Solid arrows indicate the preferred treatment pathway. Dashed arrows indicate alternative or context-dependent options.

In HER2-positive (IHC 3+ or IHC 2+/ISH+) mCRC, anti-HER2 therapy is an important option from the third line onward, particularly when rapid tumor shrinkage is needed or bevacizumab is unsuitable. Although the optimal treatment sequence has not been established, a reasonable approach in patients with *RAS* WT tumors is tucatinib plus trastuzumab (where available) or trastuzumab plus pertuzumab followed by T-DXd, particularly given T-DXd’s demonstrated activity irrespective of prior anti-HER2 therapy. Among patients with HER2-positive mCRC exhibiting low *ERBB2* copy number, irinotecan plus cetuximab represents a rational option based on the findings of the SWOG S1613 trial [113]. T-DXd is a particularly strong option for *RAS*-mutant tumors (Figure 2). By contrast, HER2-targeted therapy has not shown any clear benefit in HER2-low mCRC, and standard salvage approaches remain appropriate.

Overall, HER2-positive mCRC represents a distinct molecular subtype with effective targeted therapeutic options. However, optimal treatment sequencing will increasingly require integration of *RAS*/*BRAF* and HER2 IHC status and dynamic ctDNA-based assessment of *ERBB2* amplification.

### 4.3. KRAS G12C Inhibitors: Transforming an “Undruggable” Target

The *KRAS* G12C mutation, present in 3–4% of patients with mCRC, was historically considered “undruggable” [114,115,116,117]. However, selective inhibitors such as sotorasib and adagrasib have revolutionized the treatment of patients with this mutation. Although monotherapy has shown limited effects in these patients [118,119], the combination of these inhibitors with anti-EGFR mAbs has yielded superior outcomes compared with monotherapy by counteracting feedback-mediated pathway reactivation. The phase III CodeBreaK 300 trial demonstrated that sotorasib plus panitumumab significantly improved PFS compared with standard salvage therapies (mPFS: 5.6 vs. 2.0 months; HR: 0.48) [120].

Several other *KRAS* G12C inhibitors have achieved consistently high response rates when combined with anti-EGFR mAbs. These include garsorasib plus cetuximab (phase II; ORR: 45%; mPFS: 7.5 months) [121], divarasib plus cetuximab (phase Ib; ORR: 62%; mPFS: 8.1 months) [122], olomorasib (100 mg) plus cetuximab (phase I/II; ORR: 44%; mPFS: 7.5 months) [123], and MK-1084 plus cetuximab (KANDLELIT-001, phase I; ORR: 46%) [124].

These findings indicate that combined EGFR inhibition is a key strategy for this molecular subtype. *KRAS* G12C mutation serves as both an adverse prognostic factor and a clearly actionable biomarker in mCRC, as direct targeting can provide meaningful clinical benefit.

**Figure 3:**
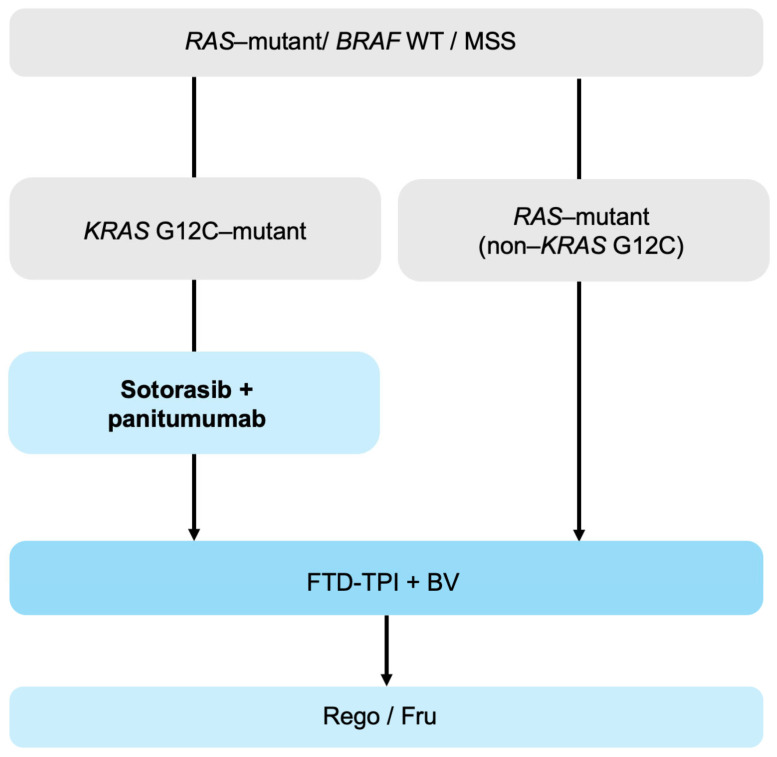
Treatment algorithm for *KRAS* G12C–mutant mCRC. Abbreviations: mCRC, metastatic colorectal cancer; WT, wild-type; MSS, microsatellite stable; FTD-TPI, trifluridine/tipiracil; BV, bevacizumab; Rego, regorafenib; Fru, fruquintinib.

In patients with *BRAF* WT, a Microsatellite Stable (MSS) disease, treatment selection is stratified by *RAS* mutation subtype. When a *KRAS* G12C mutation is detected via ctDNA, sotorasib plus panitumumab represents a rational third-line option. Given its higher response rates, this combination is often prioritized over FTD-TPI plus bevacizumab, and is considered particularly beneficial in patients requiring tumor shrinkage or those unable to receive bevacizumab. Tumors harboring *RAS* mutations other than *KRAS* G12C are generally treated with FTD-TPI plus bevacizumab. Regorafenib or fruquintinib may subsequently be used when appropriate (Figure 3). However, the optimal treatment positioning remains unclear, and further studies—especially those integrating ctDNA-guided dynamic profiling—are needed to refine sequencing strategies.

### 4.4. BRAF V600E–Mutant mCRC: From Poor Prognosis to Targeted Success

*BRAF* V600E mutations, observed in 5–10% of patients with mCRC, are associated with a highly aggressive clinical phenotype and poor prognosis [125,126,127]. Similarly to *KRAS* G12C inhibitors, BRAF inhibitors have demonstrated limited efficacy as monotherapy, necessitating combination strategies—particularly with anti-EGFR mAbs—to achieve meaningful clinical benefit. In this context, the landmark phase III BEACON CRC trial was conducted to evaluate previously treated patients with *BRAF* V600E–mutant mCRC, comparing encorafenib plus cetuximab with or without binimetinib (doublet/triplet) versus standard chemotherapy. The doublet significantly improved OS (mOS: 8.4 vs. 5.9 months; HR: 0.60; *p* < 0.001) and ORR (20% vs. 2%; *p* < 0.001) compared with the control; the triplet showed a similar OS benefit (mOS: 9.0 months; HR: 0.52) and higher ORR (26%) but only modest additional efficacy with greater toxicity. Accordingly, the doublet regimen became the clinical standard [128]. The phase III BREAKWATER trial demonstrated that early first-line treatment with encorafenib + cetuximab + mFOLFOX6 (EC + FOLFOX) in patients with untreated *BRAF* V600E–mutant mCRC significantly improved ORR (60.9% vs. 40.0%; odds ratio: 2.44; *p* = 0.0008), PFS (mPFS: 12.8 vs. 7.1 months; HR: 0.53; *p* < 0.001), and OS (mOS: 30.3 vs. 15.1 months; HR: 0.49; *p* < 0.001) compared with standard chemotherapy, thereby redefining the management of this molecular subtype [129,130].

Second-line options include FOLFIRI plus an anti-angiogenic agent (ramucirumab or aflibercept). Subgroup analyses of the RAISE and VELOUR trials revealed trends toward improved PFS and OS with angiogenesis inhibition compared with chemotherapy alone in patients with *BRAF* V600E-mutations (RAISE; mOS: 13.3 months; HR: 0.84; *p* = 0.022; mPFS: 5.7 months; HR: 0.79; *p* < 0.0005) (VELOUR; mOS: 8.9 months; HR: 0.90; *p* = 0.779; mPFS: 5.5 months; HR: 0.48; *p* = 0.062), supporting the utility of the combination therapy. For salvage-line therapies, FTD-TPI plus bevacizumab is a strong option based on findings from the SUNLIGHT trial, which demonstrated its consistent benefit regardless of *BRAF* mutation status [50,131,132,133,134].

Resistance to BRAF inhibitors can arise through MAPK pathway reactivation [135,136,137]. Informed by this mechanism, the phase II BAYONET trial is currently underway to explore the efficacy and safety of adding a MEK inhibitor to BRAF inhibitor plus anti-EGFR mAb doublet therapy in patients who have developed resistance to the doublet regimen [138]. In addition, building on experience with anti-EGFR mAb rechallenge and BRAF/MEK inhibition [139,140], Kotani et al. conducted the TRIDENTE trial to evaluate the outcomes of rechallenge with encorafenib, binimetinib, and cetuximab [141]. Conventional BRAF inhibitors may paradoxically activate MAPK signaling in RAS-mutant or WT cells via RAF dimerization, contributing to toxicity and resistance. In contrast, “paradox-breaker” BRAF inhibitors (e.g., PLX8394, PLX7904, mosperafenib) inhibit RAF dimerization and maintain MAPK suppression [142,143]. In a phase I study, mosperafenib (RG6344) demonstrated favorable tolerability without reaching the maximum tolerated dose and showed activity in BRAF inhibitor–naïve *BRAF* V600E-mutant mCRC (ORR: 25%, mPFS: 7.3 months), without typical BRAF inhibitor-related toxicities [144,145] (Table 3).

**Figure 4:**
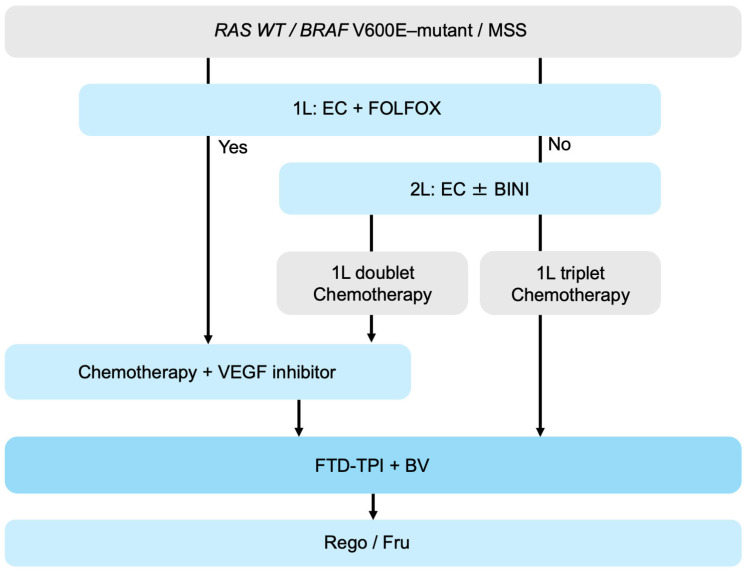
Treatment algorithm for *BRAF* V600E–mutant mCRC. Abbreviations: mCRC, metastatic colorectal cancer; WT, wild-type; MSS, microsatellite stable; EC, encorafenib plus cetuximab; BINI, binimetinib FTD-TPI, trifluridine/tipiracil; BV, bevacizumab; Rego, regorafenib; Fru, fruquintinib.

The following treatment strategies should be considered for patients with MSS, *BRAF* V600E–mutant mCRC. In the first-line setting, EC (encorafenib plus cetuximab) combined with FOLFOX is recommended. In the second-line setting, chemotherapy plus a VEGF inhibitor is generally recommended. If EC + FOLFOX was not administered in the first line, EC with or without binimetinib may be considered in the second-line setting. Among patients who received first-line cytotoxic doublet chemotherapy, third-line treatment may consist of cytotoxic chemotherapy plus a VEGF inhibitor based on the agent not previously used (oxaliplatin or irinotecan). In contrast, for patients who received first-line cytotoxic triplet chemotherapy, FTD-TPI plus bevacizumab may be considered (Figure 4). However, further evidence is needed to define the optimal sequencing for this subset of mCRCs. Adaptive sequencing strategies guided by dynamic molecular monitoring via ctDNA—enabling early detection of emerging resistant clones and treatment re-optimization based on evolving molecular profiles—are expected to be key to improving outcomes in this poor-prognosis population.

## 5. Strategies for Optimal Treatment Sequencing

The rapid expansion of the salvage-line armamentarium has shifted clinical focus toward establishing an “adaptive sequencing strategy.” Historically, the choice between regorafenib and FTD-TPI was a subject of clinical debate. The prospective OSERO study and real-world data suggest that the sequence of regorafenib followed by FTD-TPI plus bevacizumab (or vice versa) yields comparable OS, indicating that both approaches are clinically rational [146,147].

However, treatment decisions are no longer binary. The introduction of fruquintinib and molecularly guided options, such as anti-EGFR mAb rechallenge, has necessitated a more nuanced approach to treatment decision-making. Sequential therapy involving administration of fruquintinib after FTD-TPI plus bevacizumab or regorafenib is being actively evaluated, with studies such as JACCRO CC-19 designed to investigate the clinical benefit of continuous VEGF inhibition [148]. The PARERE trial highlighted that both panitumumab rechallenge and regorafenib are viable in patients with *RAS/BRAF* WT mCRC confirmed via ctDNA; however, rechallenge often provides superior ORR and PFS [149] (Table 4). Treatment selection should be guided by Eastern Cooperative Oncology Group performance status (ECOG PS), treatment goals, and toxicity profile. In patients with PS 0–1 requiring tumor shrinkage, FTD-TPI plus bevacizumab may be preferred. For patients prioritizing disease control, or those intolerant of hematologic toxicity, regorafenib or fruquintinib may be more appropriate. Prior treatment-related adverse events should also be systematically considered when determining treatment sequencing; for instance, prior proteinuria has been reported to increase the risk of subsequent proteinuria [150], underscoring the importance of a comprehensive toxicity history in clinical practice.

Ultimately, the integration of real-time molecular profiling via ctDNA into treatment algorithms represents a promising path forward, although prospective trials directly comparing sequencing strategies remain limited and the optimal treatment order continues to evolve.

## 6. Immune Checkpoint Inhibitor-Based Combination Therapy for MSS mCRC

MSS mCRC, unlike MSI-H (Microsatellite Instability-High) disease, accounts for approximately 95% of all mCRC cases but shows minimal response to immune checkpoint inhibitor (ICI) monotherapy (ORR < 5%) [6]. The phase III LEAP-017 trial (NCT04776148) evaluated the oral multikinase inhibitor lenvatinib plus pembrolizumab versus standard of care (regorafenib or FTD-TPI) in the salvage-line setting for patients with MSS mCRC. The combination did not meet the prespecified threshold for statistical significance (mOS: 9.8 vs. 9.3 months; HR: 0.83; *p* = 0.0379; prespecified threshold *p* = 0.0214) [151]. Similarly, the phase III KEYFORM-007 trial (NCT05064059) evaluated pembrolizumab combined with the anti–lymphocyte activation gene-3 (LAG-3) antibody favezelimab versus standard of care (regorafenib or FTD-TPI) in patients with MSS mCRC and PD-L1 CPS ≥ 1. This combination also failed to demonstrate an OS benefit (mOS: 7.3 vs. 8.5 months; HR: 0.98; *p* = 0.4183) [152]. However, several combination strategies have demonstrated promising activity, with the presence or absence of liver metastases emerging as a key determinant of treatment efficacy [153]. A meta-analysis of 14 trials evaluating regorafenib combined with anti-PD-1 antibodies reported modest overall efficacy (ORR: 7%; mPFS: 2.99 months). Outcomes were more favorable in patients without liver metastases (ORR: 21%; mPFS: 3.84 months) than in those with liver metastases (ORR: 6%; mPFS: 1.99 months) [154]. In a phase I trial (*n* = 39) of regorafenib combined with ipilimumab and nivolumab, the recommended phase II dose cohort achieved an ORR of 27.6% and an mOS of 20 months; notably, responses were confined to patients without liver metastases (ORR: 36.4%; mOS: 27.5 months) [155,156].

The phase III STELLAR-303 trial (NCT05425940) evaluated zanzalintinib—an oral multikinase inhibitor—in combination with the anti-PD-L1 antibody atezolizumab versus regorafenib in the salvage-line setting across 121 centers in 16 countries. The combination demonstrated a statistically significant improvement in OS (mOS: 10.9 vs. 9.4 months; HR: 0.80; *p* = 0.0045), although the clinical meaningfulness of this benefit remains subject to debate [157].

The combination of botensilimab, a next-generation CTLA-4 inhibitor, and balstilimab, an anti–PD-1 antibody, has also demonstrated promising activity. In a phase I trial involving 148 patients with MSS mCRC in the salvage-line setting, the regimen achieved an ORR of 17% and a DCR of 61%, with improved outcomes in patients without liver metastases (ORR: 22–23%; DCR: 73%) [158]. A phase III trial is currently underway (NCT07152821).

Although these approaches have not yet been incorporated into standard practice, they represent promising strategies—particularly in patients without liver metastases—that may shape future treatment paradigms.

## 7. Conclusions

The landscape of salvage-line therapy for mCRC has undergone a significant paradigm shift over time. FTD-TPI plus bevacizumab has emerged as a broadly applicable therapeutic backbone, demonstrated by the consistent improvements in PFS and OS reported in the SUNLIGHT trial. Nevertheless, a “one-size-fits-all” approach is no longer appropriate in the era of precision oncology. Real-time molecular profiling using ctDNA facilitates identification of resistance mechanisms and enables adaptation of treatment strategies based on clonal evolution. Despite these advantages, ctDNA analysis has several inherent limitations, including the lack of an optimal cutoff value, the potential for false-negative results in patients with low ctDNA shedding, confounding by CHIP, and variability across assay platforms. For patients with *RAS* WT tumors, ctDNA-guided anti-EGFR mAb rechallenge offers a rational means of restoring therapeutic sensitivity. Furthermore, identification of *ERBB2* amplification and *KRAS* G12C mutations allows for implementation of highly active targeted therapy, which should be prioritized when tumor shrinkage is a primary objective. The transition from rigid treatment algorithms to flexible, dynamic strategies driven by real-time biomarkers will be an essential evolution in clinical practice. Future prospective trials are warranted to validate these adaptive sequences and ensure that the promise of precision medicine translates into long-term survival benefits for patients with mCRC.

## Figures and Tables

**Table 1:** Clinical Trials of Conventional Salvage-Line Therapies in mCRC.

Study	Treatment Regimen	Treatment Line	Phase	Sample Size	Primary Endpoint	mOS (Months) [HR]	mPFS (Months) [HR]	ORR (%)
**CO.17 [17]**	Cet + BSC vs. BSC	≥3 L	III	572	OS	6.1 vs. 4.6 [0.77]	1.9 vs. 1.8 [0.68]	8.0 vs. 0
**20020408 [19]**	Pani + BSC vs. BSC	≥3 L	III	463	PFS	NA	2.0 vs. 1.7 [0.54]	10.0 vs. 0
**20100007 [21]**	Pani + BSC vs. BSC	≥3 L	III	377	OS	10.0 vs. 7.4 [0.70]	5.2 vs. 1.7 [0.44]	27.0 vs. 0
**BOND [22]**	Cet + Iri vs. Cet	≥3 L	II	329	ORR	8.6 vs. 6.9 [NA]	4.1 vs. 1.5 [NA]	22.9 vs. 10.8
**ASPECCT [23]**	Pani vs. Cet	≥3 L	III	1010	OS	10.4 vs. 10.0 [0.97]	4.1 vs. 4.4 [1.00]	22 vs. 19.8
**WJOG 6510G [24]**	Pani + Iri vs. Cet + Iri	≥3 L	II	121	PFS	14.9 vs. 11.5 [0.68]	5.4 vs. 4.2 [0.69]	33 vs. 28
**CORRECT [32]**	Rego vs. placebo	≥3 L	III	760	OS	6.4 vs. 5.0 [0.77]	1.9 vs. 1.7 [0.49]	1.0 vs. 0.4
**CONCUR [33]**	Rego vs. placebo	≥3 L	III	204	OS	8.8 vs. 6.3 [0.55]	3.2 vs. 1.7 [0.31]	4.0 vs. 0
**ReDOS [34]**	Dose-escalation Rego vs. standard-dose Rego	≥3 L	II	123	Proportion initiating cycle 3	9.8 vs. 6.0 [0.72]	2.5 vs. 2.0 [0.84]	0 vs. 0
**RECOURSE [36]**	FTD-TPI vs. placebo	≥3 L	III	800	OS	7.1 vs. 5.3 [0.68]	2.0 vs. 1.7 [0.48]	1.6 vs. 0.4
**TERRA [37]**	FTD-TPI vs. placebo	≥3 L	III	406	OS	7.8 vs. 7.1 [0.79]	2.0 vs. 1.8 [0.43]	1.6 vs. 0
**FRESCO [45]**	Fru vs. placebo	≥3 L	III	416	OS	9.3 vs. 6.6 [0.65]	3.7 vs. 1.8 [0.26]	4.7 vs. 0
**FRESCO-2 [46]**	Fru vs. placebo	≥3 L	III	691	OS	7.4 vs. 4.8 [0.66]	3.7 vs. 1.8 [0.32]	1.5 vs. 0
**SUNLIGHT [50]**	FTD-TPI + BV vs. FTD-TPI	≥3 L	III	492	OS	10.8 vs. 7.5 [0.61]	5.6 vs. 2.4 [0.44]	6.3 vs. 1.0
**BiTS [54]**	Biweekly FTD-TPI + BV	≥3 L	Ib/II	46	DCR	11.4	4.2	4.3
**TAS-CC4 [55]**	Biweekly FTD-TPI + BV	3 L	II	30	DCR	9.7	3.8	6.7

Abbreviations: mCRC, metastatic colorectal cancer; OS, overall survival; mOS, median overall survival; PFS, progression-free survival; mPFS, median progression-free survival; HR, hazard ratio; ORR, objective response rate; DCR, disease control rate; Cet, cetuximab; BSC, best supportive care; Pani, panitumumab; Iri, irinotecan; Rego, regorafenib; FTD-TPI, trifluridine/tipiracil; Fru, fruquintinib; BV, bevacizumab; NA, not available.

**Table 2:** Clinical Trials of Contemporary Salvage-Line Therapies in mCRC (Anti-EGFR mAb Rechallenge Trials).

Study	Treatment Regimen	Treatment Line	Phase	Sample Size	Primary Endpoint	ORR (%)	mPFS (Months) [HR]	mOS (Months) [HR]
**Santini et al. [87]**	Cet + Iri Rechallenge	≥3 L	II	39	ORR	53.8	6.6	8.2
**CRICKET [88]**	Cet + Iri Rechallenge	≥3 L	II	28	ORR	21	3.4	9.8
**E-Rechallenge [89]**	Cet + Iri Rechallenge	≥3 L	II	33	ORR	15.6	3.0	8.6
**JACCRO CC-08/09AR [90]**	Anti-EGFR mAb ± Iri	≥3 L	II	59	ctDNA *RAS*—outcome association	8.0	2.4	8.0
**CAVE [91]**	Cet + Avelumab	≥3 L	II	77	OS	7.8	3.6	11.6
**CHRONOS [93]**	Pani	≥3 L	II	27	ORR	30	3.5	7.5
**CITRIC [94]**	Cet + Iri Rechallenge vs. Rego or FTD-TPI	≥3 L	II	114	PFS	19.3 vs. 5.7	4.2 vs. 2.7 [0.66]	NA
**FIRE-4 [95]**	Cet + Iri Rechallenge vs. investigator’s choice	≥3 L	III	87	OS	26.7 vs. 11.9	5.8 vs. 4.6 [0.91]	17.6 vs. 15.1 [0.84]

Abbreviations: mCRC, metastatic colorectal cancer; ORR, objective response rate; PFS, progression-free survival; mPFS, median progression-free survival; OS, overall survival; mOS, median overall survival; HR, hazard ratio; DCR, disease control rate; Cet, cetuximab; Iri, irinotecan; Anti-EGFR mAb, Anti-EGFR monoclonal antibody; FTD-TPI, trifluridine/tipiracil; Pani, panitumumab; Rego, regorafenib; NA, not available.

**Table 3:** Clinical Trials of Contemporary Salvage-Line Therapies in mCRC (Novel Targeted Agent Trials).

Study	Treatment Regimen	Treatment Line	Phase	Sample Size	Primary Endpoint	ORR (%)	mPFS (Months) [HR]	mOS (Months) [HR]
**HERACLES-A [106]**	T-mab + Lapatinib	≥3 L	II	27	ORR	30.0	5.2	11.5
**MyPathway [107]**	Pertuzumab + T-mab	≥3 L	IIa	57	ORR	32.0	2.9	11.5
**TRIUMPH [108]**	Pertuzumab + T-mab	≥3 L	II	30	ORR	28.0	4.1	10.3
**DESTINY-CRC01 [109]**	T-DXd	≥3 L	II	53	ORR	45.3	6.9	15.5
**DESTINY-CRC02 [110]**	T-DXd (5.4 vs. 6.4 mg/kg)	≥3 L	II	122	ORR	37.8 vs. 27.5	5.8 vs. 5.5 [0.79]	13.4 vs. 11.4 [NA]
**MOUNTAINEER [111]**	Tucatinib + T-mab	≥3 L	II	84	ORR	38.1	8.2	24.1
**CodeBreaK100 [118]**	Sotorasib	≥3 L	II	62	ORR	9.7	4.0	10.6
**KRYSTAL-1 [119]**	Cet vs. Adagrasib + Adagrasib	≥3 L	I/II	94	ORR	46 vs. 19	6.9 vs. 5.6 [NA]	13.4 vs. 15.9 [NA]
**CodeBreaK300 [121]**	Sotorasib + Pani vs. FTD-TPI or Rego	≥3 L	III	160	PFS	26.0 vs. 0	5.6 vs. 2.0 [0.48]	NR vs. 10.3 [0.70]
**NCT04585035 [121]**	Cet vs. Garsorasib + Garsorasib	≥2 L	II	94	ORR	62.5 vs. 29.5	8.0 vs. 5.5 [NA]	NA
**NCT04449874 [122]**	Divarasib + Cet	≥2 L	Ib	29	Safety and tolerability	62.0	8.1	NA
**LOXO-RAS-20001** ** (NCT04956640) [123]**	Olomorasib (100 mg) + Cet vs. Olomorasib (150 mg) + Cet	≥2 L	I/II	93	Safety and tolerability	44.0 vs. 38.0	7.5 vs. 6.6 [NA]	NA
**KANDLELIT-001** ** (NCT05067283) [124]**	MK-1084 vs. MK-1084 + Cet vs. MK-1084 + Cet + mFOLFOX	≥2 L	I/II	64	Safety and tolerability	7 vs. 36.0 vs. NA	NA	NA
**BEACON CRC [128]**	ENCO + BINI + Cet vs. ENCO + Cet vs. Iri or FOLFIRI + Cet	≥2 L	III	665	OS	26 (triplet) 20 (doublet) 2 (control)	4.3 (triplet) [0.38] 4.2 (doublet) [0.40] 1.5 (control)	9.0 (triplet) [0.52] 8.4 (doublet) [0.60] 5.4 (control)

Abbreviations: mCRC, metastatic colorectal cancer; ORR, objective response rate; PFS, progression-free survival; mPFS, median progression-free survival; OS, overall survival; mOS, median overall survival; HR, hazard ratio; T-mab, trastuzumab; T-DXd, trastuzumab deruxtecan; Cet, cetuximab; Pani, panitumumab; FTD-TPI, trifluridine/tipiracil; Rego, regorafenib; ENCO, encorafenib; BINI, binimetinib; Iri, Irinotecan; NA, not available; NR, not reached.

**Table 4:** Strategies for Optimal Treatment Sequencing in mCRC.

Study	Treatment Sequence	Treatment Line	Phase	Sample Size	Primary Endpoint	mOS (Months) [HR]	mPFS (Months) [HR]
**OSERO [146]**	A: Rego → FTD-TPI B: FTD-TPI → Rego C: FTD-TPI + BV → Rego	≥3 L	NA	455	OS	11.8(A) 7.1(B) 10.2(C)	NA
**Ahn et al. [147]**	Rego → FTD-TPI ± BV vs. FTD-TPI ± BV → Rego	≥3 L	NA	343	OS	13.1 (Rego—first)	NA
**PARERE [149]**	Pani → Rego vs. Rego → Pani	≥3 L	II	112	OS	13.6 vs. 10.0 [0.67]	4.0 vs. 2.5 [0.63]

Abbreviations: mCRC, metastatic colorectal cancer; OS, overall survival; mOS, median overall survival; PFS, progression-free survival; mPFS, median progression-free survival; HR, hazard ratio; Rego, regorafenib; FTD-TPI, trifluridine/tipiracil; BV, bevacizumab; Pani, panitumumab; NA, not available.

## Data Availability

No new data were created or analyzed in this study. Data sharing is not applicable to this article.

## Abbreviations

The following abbreviations are used in this manuscript:

mCRC	Metastatic colorectal cancer
ctDNA	Circulating tumor DNA
OS	Overall survival
PFS	Progression-free survival
HR	Hazard ratio
BSC	Best supportive care
ORR	Objective response rate
WT	Wild-type
EGFR	Epidermal growth factor receptor
mAb	Monoclonal antibody
FTD-TPI	Trifluridine/tipiracil
CIN	Chemotherapy-induced neutropenia
EGFR-ECD	EGFR extracellular domain
HER2	Human epidermal growth factor receptor 2
ADC	Antibody–drug conjugate
T-Dxd	Trastuzumab Deruxtecan
CHIP	Clonal hematopoiesis of indeterminate potential
